# Sortase A-Inhibitory Metabolites from a Marine-Derived Fungus *Aspergillus* sp.

**DOI:** 10.3390/md18070359

**Published:** 2020-07-13

**Authors:** Sung Chul Park, Beomkoo Chung, Jayho Lee, Eunji Cho, Ji-Yeon Hwang, Dong-Chan Oh, Jongheon Shin, Ki-Bong Oh

**Affiliations:** 1 Natural Products Research Institute, College of Pharmacy, Seoul National University, Seoul 08826, Korea; sungchulpark@snu.ac.kr (S.C.P.); yahyah7@snu.ac.kr (J.-Y.H.); dongchanoh@snu.ac.kr (D.-C.O.); 2 Department of Agricultural Biotechnology, College of Agriculture and Life Sciences, Seoul National University, Seoul 08826, Korea; beomkoo01@snu.ac.kr (B.C.); jayho@snu.ac.kr (J.L.); eunji525@snu.ac.kr (E.C.)

**Keywords:** marine-derived fungus, *Aspergillus* sp., metabolites, sortase A, fibronectin

## Abstract

Seven alkaloidal compounds (**2**–**8**) and one polyketide (**1**) were isolated from a semisolid rice culture of the marine-derived fungus *Aspergillus* sp. F452. Structures of the isolated compounds were elucidated based on spectroscopic data and comparisons with previously reported data. The alkaloidal compounds (**2**–**8**) displayed weak to moderate inhibitory activities against *Staphylococcus aureus*-derived sortase A (SrtA) without affecting cell viability. Aspermytin A (**1**) strongly inhibited SrtA activity, with an IC_50_ value of 146.0 μM, and significantly reduced bacterial adherence to fibronectin-coated surfaces. The present results indicate that the underlying mechanism of action of compound **1** is associated with the inhibition of SrtA-mediated *S*. *aureus* adhesion to fibronectin, thus potentially serving as an SrtA inhibitor.

## 1. Introduction

Antibiotic-resistant bacteria are the most prominent limitation in conventional antimicrobial treatment [1]. Microorganisms can acquire antibiotic resistance when their survival is at risk. Whereas antibiotics have a long-standing history of success in treatment of bacterial infections, recent increasing antimicrobial resistance has stimulated the search for anti-virulence drugs as an alternative to conventional antibiotics, despite their high importance, for counteracting bacterial pathogens [2,3].

The pathogenesis of bacterial infections is initiated with bacterial adhesion to host tissue surfaces mediated via specific interactions between host ligands and bacterial surface proteins [4]. In particular, in Gram-positive bacteria including *Staphylococcus aureus*, this fundamental stage of infection proceeds through sortase-mediated anchoring of surface proteins in host cells to the bacterial cell wall envelope [5]. In *S*. *aureus*, sortase A (SrtA) cleaves surface proteins between threonine and glycine residues in LPXTG sorting signals at their C-termini and is subsequently incorporated into the bacterial cell wall envelope via a transpeptidation reaction [6,7]. Numerous knockout studies have revealed that SrtA plays a critical role in the pathogenesis of Gram-positive bacterial infections by modulating bacterial adhesion to host tissues [8,9,10]. SrtA decorates the surfaces of Gram-positive bacteria with a diverse array of proteins that enable each microbe to effectively interact with its environment and is not required for bacterial growth or viability [2,3]. It is thus considered a promising target for the development of anti-virulence drugs that aim to interfere with important virulence mechanisms, such as adhesion to host tissues.

The secondary metabolites of marine fungi, including polyketides, alkaloids, terpenes, lactones, and peptides, are a rich source of bioactive natural products [11]. Many bioactive compounds with varying degrees of action, such as antibiotic, antiviral, antimicrobial, and anticancer properties, have been isolated from marine fungal sources. Recent investigations of marine fungal metabolites looking for bioactive compounds indicate their potential as a source of new medicines [12,13]. Previously, we reported several novel natural products isolated from marine-derived fungi; polyaromatic metabolites from *Penicillium* sp. exhibited moderate cytotoxicity and significant inhibitory activity against *S*. *aureus* SrtA [14], asperphenins from *Aspergillus* sp. induced significant cytotoxicity in diverse cancer cells [15], and peptides from *A*. *allahabadii* and *A*. *ochraceopetaliformis* displayed SrtA inhibitory activity [16].

In further study, chemical investigation of *Aspergillus* sp. F452 was performed [15], whose crude extract inhibited *S*. *aureus*-derived SrtA (63% inhibition at 100 μg/mL). Bioassay-guided separation of the extract yielded seven alkaloidal (**2**–**8**) and one polyketide (**1**) compound, whose structures were analyzed through combined spectroscopic methods. This study describes the structures and biological activities of these compounds. Among them, compound **1** (aspermytin A) significantly inhibited *S*. *aureus*-derived SrtA. The in vivo bioactivity and underlying mechanism of action were also found to be associated with the inhibition of SrtA-mediated *S*. *aureus* adhesion to the eukaryotic cell matrix protein fibronectin.

## 2. Results and Discussion

### 2.1. Isolation and Structure Elucidation of Compounds ***1***–***8***

**Figure 1:**
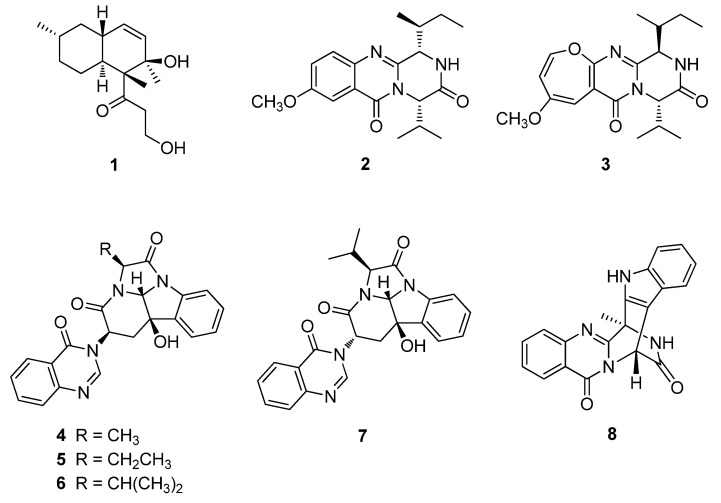
Structures of compounds **1**–**8** from *Aspergillus* sp. F452.

The fungal strain F452 [15] was cultured in semisolid rice medium and extracted with MeOH and CH_2_Cl_2_. Following solvent evaporation, the combined extract was separated by solvent partitioning followed by reversed-phase C_18_ vacuum flash chromatography and semi-preparative high performance liquid chromatography (HPLC) to yield eight compounds. Compounds **1**–**8** were identified as aspermytin A (**1**) [17], versicomide A (**2**) [18], versicoloid A (**3**) [19], isochaetominines A–C (**4**–**6**) [20,21], 14-*epi*-isochaetominine C (**7**) [21], and fumiquinazoline K (**8**) [22], respectively, via combined spectroscopic analyses, including high-resolution fast atom bombardment mass spectroscopy, ^1^H- and ^13^C-nuclear magnetic resonance (NMR), 2-D NMR, and UV spectroscopy (Figure 1). The spectroscopic data for these compounds were in good agreement with those in the literature.

### 2.2. SrtA Inhibitory Activity of Compounds ***1***–***8***

Recombinant SrtA derived from *S*. *aureus* ATCC6538p was purified from *Escherichia coli* extracts using metal chelate-affinity chromatography [23]. The enzyme activity was determined from the fluorescence intensity upon cleavage of a peptide substrate containing the LPETG motif [24]. Throughout the separation process, the crude extract and chromatographic fractions containing fungal metabolites of strain F452 inhibited the activity of SrtA. Accordingly, the same bioassay was performed using pure compounds. The inhibitory potencies of the pure compounds against recombinant SrtA, expressed as IC_50_ values, are shown in Table 1 and are compared to those of the known SrtA inhibitors berberine chloride (IC_50_ = 85.9 μM) and *para*-hydroxymercuribenzoic acid (*p*HMB) (IC_50_ = 112.5 μM). The pure compounds **1**–**8** displayed weak to significant SrtA inhibition (IC_50_ values of 269.4–146.0 μM). Among them, compound **1** exhibited the most potent inhibitory activity.

**Figure 2:**
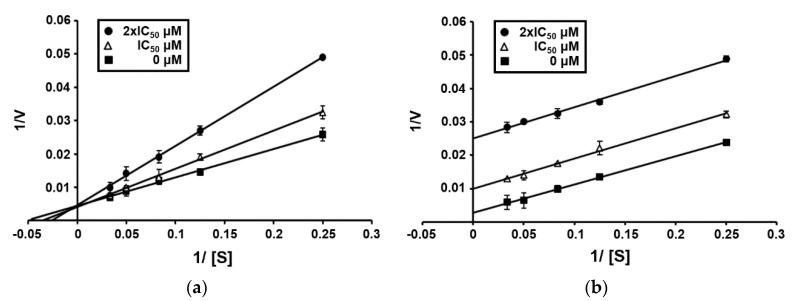
Lineweaver–Burk plot of SrtA inhibition by compounds **1** (**a**) and **3** (**b**). [S], substrate concentration [μM]; *V*, reaction velocity (Δabsorbance unit/min). Each data point represents the mean of three experiments.

To determine the type of inhibition, kinetic studies were performed with compounds **1** and **3** at IC_50_ or twofold IC_50_ based on a Lineweaver and Burk plot [25] (Figure 2). Inhibitor constants were obtained by a Dixon plot. Inhibitory kinetics show that compound **1** behaved as a mixed inhibitor (*K*i = 265.0 μM). In contrast, compound **3** behaved as an uncompetitive inhibitor (*K*i = 83.0 μM). Moreover, the binding of compounds **1** and **3** to SrtA was reversible because the enzyme activity was indeed recovered by dialysis within 2 h, excluding the possible existence of a covalent bond between inhibitor and enzyme.

### 2.3. Antibacterial Activity and Cytotoxicity of Compounds ***1***–***8***

Because SrtA inhibitors are expected to serve as anti-infective agents and inhibit bacterial pathogenesis without affecting cell viability [8], the minimum inhibitory concentrations (MICs) of these compounds were also measured to exclude the possible effects of test compounds on *S*. *aureus* cell adhesion to the eukaryotic cell matrix protein fibronectin owing to the inhibition of cell growth. The compounds did not exhibit inhibitory activity against *S*. *aureus* ATCC6538p (MIC > 128 μg/mL) (Table 1). In the cytotoxicity assay against A549 (lung cancer) and K562 (leukemia) cell lines, compounds **1**–**8** displayed weak (**2**–**7**: IC_50_ > 13–50 μM) to no inhibitory activity (**1** and **8**: IC_50_ > 100 μM), comparable to etoposide (IC_50_ = 0.5 μM).

### 2.4. Inhibition of SrtA-mediated S. aureus Adhesion to Fibronectin by Compound ***1***

**Figure 3:**
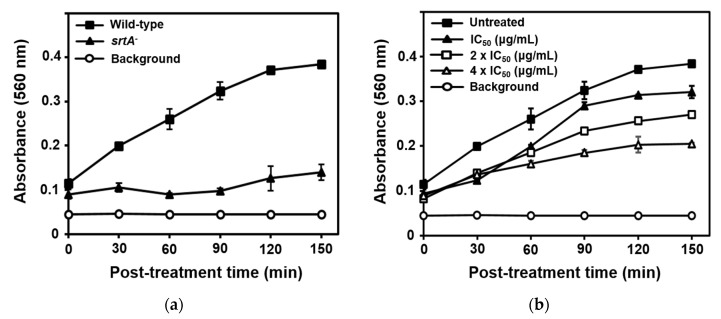
Adhesion of *S*. *aureus* strain Newman (wild-type) and the isogenic *srtA* knockout mutant (*srtA*^−^) to fibronectin (**a**), and inhibition of Newman strain adhesion to fibronectin by compound **1** (**b**) with 0×, 1×, 2×, or 4× the SrtA IC_50_ value. The results are presented as the mean ± standard deviation of three replicates.

An active SrtA enzyme is required for the attachment of *S*. *aureus* to eukaryotic cell matrix proteins, such as fibronectin and fibrinogen, thus accelerating bacterial adhesion to host tissues and subsequent invasion [26]. The *srtA*^−^ mutant strain cannot bind these proteins. Thus, SrtA inhibitors should inhibit SrtA activity in vivo and in turn reduce fibronectin-binding protein surface display. Initially, the SrtA-mediated fibronectin-binding capacities of *S*. *aureus* strain Newman (wild-type) and its isogenic *srtA* knockout mutant (*srtA*^−^) to fibronectin were evaluated. As shown in Figure 3a, the fibronectin-binding activity of the *srtA* knockout mutant was significantly reduced compared to that of the wild type. Based on SrtA inhibition intensity, compound **1** was selected. The results of the inhibition of *S*. *aureus* adhesion to fibronectin via fibronectin-binding protein by compound **1** are shown in Figure 3b. As expected, treatment of strain Newman with 0-, 1-, 2-, or 4-fold the SrtA IC_50_ of compound **1** significantly reduced bacterial adherence to fibronectin-coated surfaces. The onset and magnitude of inhibition of Newman strain adhesion to fibronectin by compound **1** with 4× the SrtA IC_50_ value was comparable to the behavior of the untreated *srtA* knockout mutant, as shown in Figure 3a. The results of the fibronectin-binding assay suggested the potential of this compound in treating *S*. *aureus* infections through inhibition of SrtA activity.

## 3. Materials and Methods

### 3.1. General Experimental Procedures

The UV spectra were acquired with a Hitachi U-3010 spectrophotometer (Tokyo, Japan). The NMR spectra were recorded in DMSO-*d*_6_ solution using Bruker Avance (400, 500, or 600) instruments (Billerica, MA, USA). IR spectra were recorded on a JASCO 4200 FT-IR spectrometer (Easton, MD, USA) using a ZnSe cell. High-resolution FABMS data were acquired using a JEOL JMS 700 mass spectrometer (Tokyo, Japan) with 6 keV-energy, emission current 5.0 mA, xenon as inert gas, and meta-nitrobenzyl alcohol (NBA) as the matrix at the Korea Basic Science Institute (Daegu, Korea). Low-resolution ESIMS data were recorded on an Agilent Technologies 6130 quadrupole mass spectrometer with an Agilent Technologies 1200 series HPLC system (Santa Clara, CA, USA). HPLC analyses were performed on a Spectrasystem p2000 equipped with a Spectrasystem RI-150 refractive index detector (Waltham, MA, USA). All of the solvents used were spectroscopic grade or distilled from glass prior to use.

### 3.2. Fungal Material

The isolation and identification of *Aspergillus* sp. (strain number F452) have previously been reported [15]. The fungal strain was isolated from submerged, decaying wood off the shore of Jeju Island, Korea, and identified using standard molecular biological protocols by DNA amplification and sequencing of the ITS region. The nucleotide sequence of F452 has been deposited in the GenBank database under accession number KF384188.

### 3.3. Extraction and Isolation

The isolated strain was cultivated on a YPG agar plate (5 g yeast extract, 5 g peptone, 10 g glucose, and 16 g agar in 1 L artificial seawater) for 4 days. The agar plugs (1 cm × 1 cm, 5 pieces each) were inoculated into 100 mL YPG media in a 250 mL Erlenmeyer flask for 5 days, then separately transferred to 2.8 L glass Fernbach flasks with rice media (200 g rice, 2 g peptone, and 2 g yeast extract with 200 mL artificial seawater in each flask, boiled in an autoclave for 20 min at 120 °C; 50 flasks in total).

Fermentation in rice media was conducted under static conditions for 6 weeks followed by extraction of each flask with MeOH (1 L × 3) and CH_2_Cl_2_ (1 L × 3). The solvent was combined and evaporated to obtain an organic extract. The combined extracts (247.33 g) were successively partitioned between *n*-BuOH (177.12 g) and H_2_O (70.05 g); the former fraction was repartitioned using H_2_O-MeOH (15:85) (83.31 g) and *n*-hexane (93.18 g). The H_2_O-MeOH fraction was separated by C_18_ reversed-phase vacuum flash chromatography using a sequential mixture of MeOH and H_2_O as eluents (five fractions in the gradient, H_2_O-MeOH, from 60:40 to 0:100), acetone, and finally EtOAc.

Based on the results of ^1^H NMR analyses and bioactivity tests, the 30:70 H_2_O-MeOH fraction (9.20 g) was separated by semi-preparative reversed-phase HPLC (YMC-ODS-A column, 10 × 250 mm; H_2_O-MeOH, 50:50; 1.7 mL/min) and yielded compounds **1** (*t*_R_ = 46.1 min), **2** (*t*_R_ = 22.2 min), **3** (*t*_R_ = 13.5 min), **4** (*t*_R_ = 37.2 min), **5** (*t*_R_ = 40.5 min), **6** (*t*_R_ = 66.9 min), **7** (*t*_R_ = 74.1 min), and **8** (*t*_R_ = 15.4 min). Compounds **1**, **4**, and **5** were further purified by analytical HPLC (YMC-ODS-A column, 4.6 × 250 mm; H_2_O-MeCN, 65:35; 0.7 mL/min; *t*_R_ = 18.2, 11.9, and 14.5 min, respectively). The purified metabolites were isolated at the following yields: 5.5, 3.4, 4.4, 2.9, 5.1, 9.3, 1.9, and 7.9 mg for **1**–**8**, respectively.

### 3.4. SrtA Inhibition Assay

The *srtA* gene from *S*. *aureus* ATCC6538p was expressed and recombinant SrtA was purified as previously described [23]. The SrtA inhibition test was carried out by analyzing the increased fluorescence intensity resulting from the cleavage of synthetic peptide substrate dabcyl-LPETG-edans (AnaSpec, Inc., Fremont, CA, USA) [9,24] with slight modification. The reaction was carried out with 100 μL buffer (50 mM Tris-HCl, 5 mM CaCl_2_, and 150 mM NaCl, pH 7.5), 7.5 µM synthetic peptide, 7.5 µM purified SrtA, and test samples at various concentrations. Each sample was dissolved in dimethyl sulfoxide (DMSO) and diluted with reaction buffer to obtain a final concentration of 1% DMSO, which did not influence enzyme activity. The SrtA inhibition assay was conducted at 37 °C for 1 h, and inhibition was quantified fluorometrically using a microplate reader (FLx800, BioTek Instruments, Winooski, VT, USA) at excitation and emission wavelengths of 350 and 495 nm, respectively. *p*HMB and berberine chloride were used as reference inhibitors of SrtA.

### 3.5. Enzyme Kinetics

All sortase (SrtA) assays were performed at 37 °C in SrtA enzyme buffer as described above. The inhibitors **1** and **3** were dissolved in DMSO and immediately diluted to the desired working concentration with the same SrtA buffer. The enzymatic inhibition measurements were carried out at different substrate concentrations in the presence and absence of a given concentration of inhibitor, and their kinetics were evaluated by the Lineweaver and Burk plot method [25]. For the dialysis kinetic studies, a solution of enzyme (0.96 mL, 75 μM) and fixed inhibitor concentration (0.04 mL) was prepared and dialyzed against 100 mL buffer at 37 °C for 2 h using regenerated cellulose dialysis membranes SPECTRAPOR^®^ (Rancho Dominguez, CA, USA). Aliquots of 100 μL of the enzyme-inhibitor mixture were taken in time intervals of 0, 30, 60, 90, and 120 min and added to 0.020 mL of substrate (7.5 μM), and after an hour of incubation the enzyme activity was measured. A control solution prepared with enzyme and buffer (SrtA buffer, 100 μL; SrtA, 7.5 μM) was treated similarly.

### 3.6. Antibacterial Activity Assay

The MICs of test compounds were determined as previously described [27]. *S*. *aureus* ATCC6538p (5 mL) was cultured in tryptic soy broth to saturation at 37 °C and diluted to an OD_600_ of 0.01. The culture was incubated for an additional 2 h and diluted to an OD_600_ of 0.005. In each well of a 96-well plate, 180 µL of cells was mixed with 20 µL of a concentrated test compound solution in 10% DMSO (final concentration, 1% DMSO). Culture plates were incubated overnight at 37 °C, and the OD_600_ was measured using a Multiskan Spectrum spectrophotometer (Thermo Labsystems Inc., Beverly, MA, USA). MIC values were defined as the lowest concentration of the test compounds inhibiting cell growth. Ampicillin was used as a positive control.

### 3.7. Cytotoxicity Assay

The effect of compounds (**1**–**8**) on cell proliferation was measured by the sulforhodamine B (SRB) cellular protein-staining method [28]. In brief, A549 (lung cancer) and K562 (leukemia) cells (1 × 10^4^ cells in 190 μL of complete DMEM) were seeded in 96-well plates with various concentrations of compounds (**1**–**8**) and incubated at 37 °C in a humidified atmosphere with 5% CO_2_. After 72 h of compound (**1**–**8**) treatment, the cells were fixed with 10% TCA solution for 1 h, and cellular proteins were stained with a solution of 0.4% SRB in 1% acetic acid. The stained cells were dissolved in 10 mM Tris buffer (pH 10.0). The effect of compounds (**1**–**8**) on cell viability was calculated as a percentage relative to a solvent-treated control, and the IC_50_ values were calculated using a nonlinear regression analysis (percent survival versus concentration). Etoposide was used as a positive control.

### 3.8. Fibronectin-Binding Assay

*S*. *aureus* strains used were Newman (wild-type) and the isogenic *srtA* knockout mutant (*srtA^−^*) [9]. These strains were cultured in tryptic soy broth at 37 °C at 200 rpm up to mid-log phase (OD_600_ = 0.5). The fibronectin-binding assay was performed as described previously [24,29]. Cells were treated with test compounds at their indicated concentrations. Every 30 min for 2.5 h, a 0.65 mL cell suspension was centrifuged at 10,000× *g* for 10 min, and the supernatant was eliminated. Following incubation overnight at −20 °C, pellets were resuspended in 0.65 mL phosphate-buffered saline (PBS) and distributed as a 100 µL scale in fibronectin-coated flat-bottomed 96-well plates (Corning Life Sciences, Tewksbury, MA, USA). The cell suspension was removed and washed with PBS following incubation at 37 °C for 2 h. Bound cells were fixed via incubation with 2% (*v*/*v*) glutaraldehyde for 30 min. After a second wash with PBS, cells were stained for 15 min with 100 μL crystal violet dye (12.5 g/L). Each well was washed with PBS and covered with aluminum foil. Plates were dried overnight and absorbance was measured at 560 nm using a microplate reader.

## 4. Conclusions

Seven alkaloidal compounds (**2**–**8**) and one polyketide (**1**) were isolated from a semisolid rice culture of the marine-derived fungus *Aspergillus* sp. F452. The structures of these compounds were obtained through a combination of spectroscopic analyses, and their data were in good agreement with previous reports. Bioactivity studies have revealed that compound **1** from a marine-derived fungus *Aspergillus* sp., separated from the mussel *Mytilus edulis*, shows significant neurotrophic effects on PC-12 cells [17]. Compound **3** from the deep sea-derived *A*. *versicolor* SCSIO 05,879 exhibits antifungal activity against the phytopathogenic fungus *Colletotrichum acutatum* (minimum inhibitory concentration (MIC) of 1.6 μg/mL) [19]. Compounds **4**–**7** from a marine-derived fungus *Aspergillus* sp. F452 exhibit weak inhibition against Na^+^/K^+^-ATPase (IC_50_ values of 20–78 μM) [20]. Compound **6** also displays weak inhibition against *Bacillus subtilis*, and compound **7** from *A*. *fumigatus*, an endophytic fungus, shows weak cytotoxic activity against the human prostate cancer cell line PC3 [21]. In our measurement of SrtA enzyme activity, compounds **1**–**8** displayed moderate to significant SrtA inhibition, comparable to berberine chloride and *p*HMB, against *S*. *aureus*-derived SrtA, a transpeptidase responsible for anchoring surface proteins to the peptidoglycan cell wall in Gram-positive bacteria. Further bioassays of compound **1** indicated that the underlying mechanism of action was associated with the inhibition of adhesion of *S*. *aureus* to fibronectin via fibronectin-binding protein. Our results demonstrate the potential of these metabolites for the development of new agents to treat Gram-positive bacterial infections by inhibiting SrtA activity.

## Figures and Tables

**Table 1:** Inhibitory activity of compounds **1**–**8** toward the activity of the SrtA enzyme and bacterial growth of *S*. *aureus* ATCC6538p.

Compounds	SrtA IC_50_ μM (μg/mL)	MIC μM (μg/mL) ^1^
**1**	146.0 ± 2.3 (38.9 ± 0.6)	>480.5 (>128)
**2**	269.4 ± 3.9 (92.5 ± 1.4)	>372.7 (>128)
**3**	193.5 ± 2.5 (69.5 ± 0.9)	>356.1 (>128)
**4**	267.9 ± 4.1 (107.8 ± 1.5)	>318.1 (>128)
**5**	232.5 ± 3.7 (96.8 ± 1.4)	>307.4 (>128)
**6**	216.4 ± 2.5 (93.1 ± 1.1)	>297.4 (>128)
**7**	237.1 ± 3.8 (102.1 ± 1.2)	>297.4 (>128)
**8**	235.1 ± 2.6 (83.8 ± 0.9)	>359.2 (>128)
Berberine chloride	85.9 ± 1.2 (31.9 ± 0.4)	>332.2 (>128)
*p*HMB	112.5 ± 1.7 (33.1 ± 0.5)	ND ^2^
Ampicillin	ND	0.4 (0.1)

^1^ MIC means minimum inhibitory concentration. ^2^ ND means not determined. *p*HMB (*para*-hydroxymercuribenzoic acid) and berberine chloride were used as reference inhibitors of SrtA. Ampicillin was used as a standard antibacterial drug.

## References

[1] Gould I.M. (2009). Antibiotic resistance: The perfect storm. Int. J. Antimicrob Agents.

[2] Maresso A.W., Schneewind O. (2008). Sortase as a target of anti-infective therapy. Pharm. Rev..

[3] Cascioferro S., Totsika M., Schillaci D. (2014). Sortase A: An ideal target for anti-virulence drug development. Microb. Pathog..

[4] Rasko D.A., Sperandio V. (2010). Anti-virulence strategies to combat bacteria-mediated disease. Nat. Rev. Drug. Discov..

[5] Hendrickx A.P., Budzik J.M., Oh S.Y., Schneewind O. (2011). Architects at the bacterial surface-sortases and the assembly of pili with isopeptide bonds. Nat. Rev. Microbiol..

[6] Mazmanian S.K., Skaar E.P., Gaspar A.H., Humayun M., Gornicki P., Jelenska J., Joachmiak A., Missiakas D.M., Schneewind O. (2003). Passage of heme-iron across the envelope of Staphylococcus aureus. Science.

[7] Clancy K.W., Melvin J.A., McCafferty D.G. (2010). Sortase transpeptidases: Insights into mechanism, substrate specificity and inhibition. Biopolymers.

[8] Mazmanian S.K., Liu G., Jensen E.R., Lenoy E., Schneewind O. (2000). Staphylococcus aureus sortase mutants defective in the display of surface proteins and in the pathogenesis of animal infections. Proc. Natl. Acad. Sci. USA.

[9] Mazmanian S.K., Ton-That H., Su K., Schneewind O. (2002). An iron-regulated sortase anchors a class of surface protein during Staphylococcus aureus pathogenesis. Proc. Natl. Acad. Sci. USA.

[10] Weiss W.J., Lenoy E., Murphy T., Tardio L., Burgio P., Projan S.J., Schneewind O., Alksne L. (2004). Effect of srtA and srtB gene expression on the virulence of Staphylococcus aureus in animal models of infection. J. Antimicrob. Chemother.

[11] Rateb M.E., Ebel R. (2011). Secondary metabolites of fungi from marine habitats. Nat. Prod. Rep..

[12] Jin L., Quan C., Hou X., Fan S. (2016). Potential pharmacological resources: Natural bioactive compounds from marine-derived fungi. Mar. Drugs.

[13] Youssef F.S., Ashour M.L., Singab A.N.B., Wink M. (2019). A comprehensive review of bioactive peptides from marine fungi and their biological significance. Mar. Drugs.

[14] Julianti E., Lee J.-H., Liao L., Park W., Park S., Oh D.-C., Oh K.-B., Shin J. (2013). New polyaromatic metabolites from a marine-derived fungus *Penicillium* sp.. Org. Lett..

[15] Liao L., Bae S.Y., Won T.H., You M., Kim S.-H., Oh D.-C., Lee S.K., Oh K.-B., Shin J. (2017). Asperphenins A and B, lipopeptidyl benzophenones from a marine-derived *Aspergillus* sp. fungus. Org. Lett..

[16] Hwang J.-Y., Lee J.-H., Park S.C., Lee J., Oh D.-C., Oh K.-B., Shin J. (2019). New peptides from the marine-derived fungi *Aspergillus* allahabadii and *Aspergillus* ochraceopetaliformis. Mar. Drugs.

[17] Tsukamoto S., Miura S., Yamashita Y., Ohta T. (2004). Aspermytin A: A new neurotrophic polyketide isolated from a marine-derived fungus of the genus Aspergillus. Bioorg. Med. Chem. Lett..

[18] Pan C., Shi Y., Chen X., Chen C.-T.A., Tao X., Wu B. (2017). New compounds from a hydrothermal vent crab-associated fungus Aspergillus versicolor XZ-4. Org. Biomol. Chem..

[19] Wang J., He W., Huang X., Tian X., Liao S., Yang B., Wang F., Zhou X., Liu Y. (2016). Antifungal new oxepine-containing alkaloids and xanthones from the deep-sea-derived fungus Aspergillus versicolor SCSIO 05879. J. Agric. Food Chem..

[20] Liao L., You M., Chung B.K., Oh D.-C., Oh K.-B., Shin J. (2015). Alkaloidal metabolites from a marine-derived *Aspergillus* sp. fungus. J. Nat. Prod..

[21] Xie F., Li X.-B., Zhou J.-C., Xu Q.-Q., Wang X.-N., Yuan H.-Q., Lou H.-X. (2015). Secondary metabolites from *Aspergillus fumigatus*, an endophytic fungus from the liverwort Heteroscyphus tener (Steph) Schiffn. Chem. Biodivers..

[22] Heredia M.L., de la Cuesta E., Avendano C. (2002). Acid-promoted reactions in 1-hydroxy, 1-dimethylaminomethyl and 1-methylene-4-arylmethyl-2,4-dihydro-1H-pyrazino[2,1-b]-quinazoline-3,6-diones. Tetrahedron.

[23] Oh K.-B., Kim S.-H., Lee J., Cho W.-J., Lee T., Kim S. (2004). Discovery of diarylacrylonitriles as a novel series of small molecule sortase A inhibitors. J. Med. Chem..

[24] Oh K.-B., Oh M.-N., Kim J.-G., Shin D.-S., Shin J. (2006). Inhibition of sortase-mediated *Staphylococcus aureus* adhesion to fibronectin via fibronectin-binding protein by sortase inhibitors. Appl. Microbiol. Biotechnol..

[25] Lineweaver H., Burk D. (1934). The determination of enzyme dissociation constants. J. Am. Chem. Soc..

[26] Alksne L.E., Projan S.J. (2000). Bacterial virulence as a target for antimicrobial chemotherapy. Curr. Opin. Biotechnol..

[27] Frankel B.A., Bentley M., Kruger R.G., McCafferty D.G. (2004). Vinyl sulfones: Inhibitors of srtA, a transpeptidase required for cell wall protein anchoring and virulence in Staphylococcus aureus. J. Am. Chem. Soc..

[28] Kim T.S., Shin Y.-H., Lee H.-M., Kim J.K., Choe J.H., Jang J.-C., Um S., Jin H.S., Komatsu M., Cha G.-H. (2017). Ohmyungsamycins promote antimicrobial responses through autophagy activation via AMP-activated protein kinase pathway. Sci. Rep..

[29] Elgalai I., Foster H.A. (2003). Comparison of adhesion of wound isolates of Staphylococcus aureus to immobilized proteins. J. Appl. Micrbiol..

